# α-Adrenergic Blockade Unmasks a Greater Compensatory Vasodilation in Hypoperfused Contracting Muscle

**DOI:** 10.3389/fphys.2012.00271

**Published:** 2012-07-18

**Authors:** Darren P. Casey, Michael J. Joyner

**Affiliations:** ^1^Human and Integrative Physiology Laboratory, Department of Anesthesiology, Mayo ClinicRochester, MN, USA

**Keywords:** alpha-adrenergic receptors, vascular resistance, blood flow, exercise, hypoperfusion

## Abstract

We previously demonstrated that acute hypoperfusion in exercising human muscle causes an immediate increase in vascular resistance that is followed by a partial restoration (less than 100% recovery) of flow. In the current study we examined the contribution of α-adrenergic vasoconstriction in the initial changes in vascular resistance at the onset of hypoperfusion as well as in the recovery of flow over time. Nine healthy male subjects (29 ± 2) performed rhythmic forearm exercise (20% of maximum) during hypoperfusion evoked by intra-arterial balloon inflation. Each trial included; baseline, exercise prior to inflation, exercise with inflation, and exercise after deflation (3 min each). Forearm blood flow (FBF; ultrasound), local (brachial artery), and systemic arterial pressure (MAP; Finometer) were measured. The trial was repeated during phentolamine infusion (α-adrenergic receptor blockade). Forearm vascular conductance (FVC; ml min^−1^ 100 mmHg^−1^) and resistance (mmHg ml min^−1^) was calculated from BF (ml min^−1^) and local MAP (mmHg). Recovery of FBF and FVC (steady state inflation plus exercise value − nadir)/[steady state exercise (control) value − nadir] with phentolamine was enhanced compared with the respective control (no drug) trial (FBF = 97 ± 5% vs. 81 ± 6%, *P* < 0.05; FVC = 126 ± 9% vs. 91 ± 5%, *P* < 0.01). However, the absolute (0.05 ± 0.01 vs. 0.06 ± 0.01 mmHg ml min^−1^; *P* = 0.17) and relative (35 ± 5% vs. 31 ± 2%; *P* = 0.41) increase in vascular resistance at the onset of balloon inflation was not different between the α-adrenergic receptor inhibition and control (no drug) trials. Therefore, our data indicate that α-adrenergic mediated vasoconstriction restricts compensatory vasodilation during forearm exercise with hypoperfusion, but is not responsible for the initial increase in vascular resistance at the onset of hypoperfusion.

## Introduction

In animals, when blood flow is restricted and/or perfusion pressure is reduced, the active muscle is capable of autoregulating its blood flow (Stainsby, 1962; Jones and Berne, 1964; Britton et al., 1985; Metting et al., 1986) via intrinsic control mechanisms (Jones and Berne, 1964; Britton et al., 1985). Additionally, a reflex pressor response contributes to the restoration of blood flow to under perfused exercising muscle in dogs (Wyss et al., 1983; Sheriff et al., 1987; Mittelstadt et al., 1994; O’leary and Sheriff, 1995; Laprad et al., 1999). Using a novel balloon catheter model in the brachial artery to reduce blood flow to contracting forearm muscles, we previously demonstrated that local vasodilator and/or myogenic mechanisms, rather than a pressor response, are responsible for a substantial portion of the restoration of flow to hypoperfused exercising human muscle (Casey and Joyner, 2009b). In subsequent studies we found that nitric oxide- and adenosine-, but not prostaglandin-, mediated vasodilation play a substantial role in the compensatory flow response to hypoperfusion (Casey and Joyner, 2009a, 2011a,c).

Collectively, our series of studies (Casey and Joyner, 2009a,b, 2011a,c) demonstrated that there is only partial compensation of flow (<100% recovery) in response to local reductions in oxygen availability via hypoperfusion. This is in contrast to other conditions in which oxygen availability is decreased (e.g., hypoxia and anemia). Under these conditions there is a compensatory vasodilation that mirrors the decrease in oxygen content (Gonzalez-Alonso et al., 2006; Wilkins et al., 2006; Casey et al., 2010). Interestingly, our hypoperfusion studies also revealed an initial increase in vascular resistance in the forearm at the onset of balloon inflation that progressively decreases over the inflation period. The initial rise in vascular resistance in these studies is in contrast to the classic view of autoregulation (a decrease in perfusion pressure is followed by a reduction in resistance to blood flow through the muscle; Stainsby, 1962; Granger et al., 1976; Britton et al., 1985; Ping and Johnson, 1992). However, the initial increase in vascular resistance is not unprecedented in that an immediate increase in vascular resistance has also been reported to occur in isolated perfused skeletal muscle of dogs (Jones and Berne, 1964). The reason for the initial increase in vascular resistance at the onset of balloon inflation and the incomplete restoration over time is not fully understood. Of interest to the current study, Daley et al. (2003) demonstrated that an increase in muscle sympathetic nerve activity (MSNA) can blunt the restoration of flow to hypoperfused exercising muscle induced by external positive pressure. An increased sympathetic restraint in the microcirculation distal to a stenosis of a proximal artery may have clinical implications as it suggests that conditions with elevated MSNA might be more susceptible to hypoperfusion and ischemia during exercise. Therefore, the aim of this study was to examine the contribution of α-adrenergic vasoconstriction in the initial changes in vascular resistance at the onset of hypoperfusion as well as in the recovery of flow over time.

## Materials and Methods

### Subjects

A total of nine young healthy male subjects volunteered to participate in the study. Subjects gave written informed consent and were non-obese, non-smokers, and were not taking any medications. Studies were performed after an overnight fast and after the subjects refrained from exercise and caffeine for at least 24 h. All study protocols were approved by Institutional Review Board and in accordance with the *Declaration of Helsinki*. Other data collected in this set of subjects has been previously reported by our group (Casey and Joyner, 2009b).

### Heart rate and systemic blood pressure

Heart rate (HR) was measured by three-lead electrocardiography (ECG). Systemic blood pressure was assessed (beat-to-beat) with a finger plethysmograph (Finometer) on the non-exercising hand and verified with an automated cuff on the same arm. The systemic pressure was used as an index of pressure proximal (upstream) from the balloon. Cardiac output (CO) was estimated using the Modelflow technique which has been validated against other techniques and used in exercise studies (Wesseling et al., 1993; Ogoh et al., 2003).

### Arterial catheterization and balloon placement

Brachial catheter placement and balloon insertion has been described in detail previously (Casey and Joyner, 2009b). Briefly, a 20-gage, 5 cm catheter was placed in the brachial artery in the experimental arm using ultrasound guidance under aseptic conditions after local anesthesia (2% lidocaine). A guide wire was then placed in the artery which was then cannulated with a four-French introducer (Cook Inc., Bloomington, IN, USA) that permitted insertion of a two-French Fogarty balloon catheter into the brachial artery. A port and stopcock system allowed the measurement of arterial pressure, administration of study drugs and drawing of arterial blood samples. The system was continuously flushed (3 ml h^−1^) with heparinized saline. The configuration of the balloon upstream from the lumen of the introducer allowed measurement of the arterial pressure distal to the balloon that was perfusing the contracting forearm muscles.

### Forearm blood flow

Brachial artery mean blood velocity (MBV) and brachial artery diameter were determined with a 12 MHz linear-array Doppler probe (Model M12L, Vivid 7, General Electric, Milwaukee, WI, USA). Brachial artery blood velocity was measured throughout each condition with a probe insonation angle previously calibrated to 60°. Brachial artery and balloon diameter measurements were obtained at end diastole and between contractions during steady-state conditions. Diameter measurement typically results in the loss of the pulse wave signal for 15–20 s. Therefore, brachial artery diameters were not obtained for the target balloon inflation (nadir) and the first 10 s immediately following balloon deflation (acute) portions of the trial. The diameters obtained immediately prior to each of these time points were used to calculate blood flow. It should be noted that we have previously demonstrated that the brachial artery diameter does not change in response to inflation and deflation of the balloon at rest and during exercise (Casey and Joyner, 2009b). Velocity and diameter measurements were made 2–3 cm proximal to the balloon. FBF was calculated as the product of MBV (cm s^−1^) and brachial artery cross-sectional area (cm^2^) and multiplied by 60 to present as milliliters per minute.

### Forearm exercise

Rhythmic forearm exercise was performed with a hand grip device by the non-dominant arm at 20% of each subject’s maximal voluntary contraction (MVC, mean 49 ± 2 kg, range 43–58 kg). The weight was lifted 4–5 cm over a pulley at a duty cycle of 1 s contraction/and 2 s relaxation (20 contractions per min) using a metronome to insure correct timing. The average weight used for forearm exercise was 9.9 ± 0.4 kg.

### Brachial artery balloon inflation

To reduce FBF the brachial artery was partially occluded via inflation of the Fogarty balloon catheter with saline using a calibrated microsyringe for tight control of balloon volume. Balloon inflations were targeted to reduce MBV by 40–50%.

### Pharmacological infusions

Phentolamine, a non-selective α-adrenergic antagonist, was administered to the exercising forearm via brachial artery catheter as a loading dose [10 μg (dl forearm volume)^−1^ min^−1^ for 5 min] followed by a continuous maintenance dose (50 μg min^−1^). To confirm α-adrenergic receptor blockade, tyramine was administered [12 μg (dl forearm volume)^−1^ min^−1^ for 3 min] to evoke endogenous norepinephrine release and stimulate both α_1_- and α_2_-adrenergic receptors before and after phentolamine (Frewin and Whelan, 1968; Dinenno et al., 2002a,b).

### Experimental protocol

**Figure 1:**
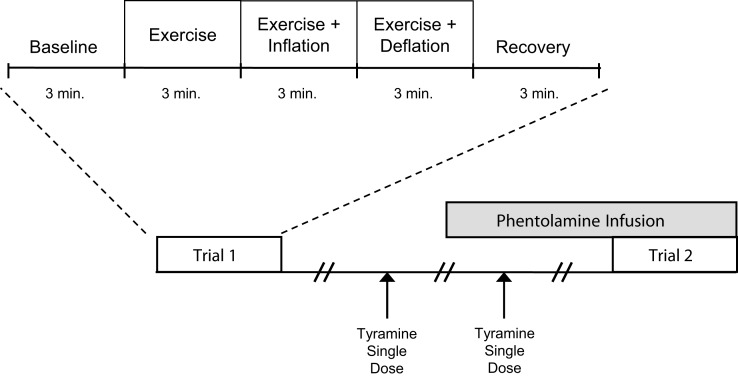
**Schematic diagram of experimental protocol**. Subjects completed two trials. Each trial consisted of baseline, exercise (control), exercise during inflation, exercise following deflation, and recovery measurements (3 min each). Trials were performed during control (no drug) and α-adrenergic receptor blockade (phentolamine). Each trial was separated by at least 20 min of rest to allow FBF to return to baseline values. Tyramine was infused with and without phentolamine to confirm the effectiveness of α-adrenergic blockade.

A schematic of the general experimental design is illustrated in Figure 1. Each subject completed a control (no drug) and an α-adrenergic blockade (phentolamine) trial. Each trial consisted of 3 min of rest, exercise, exercise with balloon inflation, exercise following balloon deflation, and recovery (15 min total; 9 min of total exercise). The phentolamine trials were always performed last due to the drug’s half-life. Each trial was separated by 20 min of rest to allow FBF to return to baseline.

### Data analysis and statistics

Data were collected at 200 Hz, stored on a computer and analyzed off-line with signal processing software (WinDaq, DATAQ Instruments, Akron, OH, USA). Local mean arterial pressure (BAP) was determined from the brachial artery pressure waveform measured distal to the balloon, systemic MAP (e.g., pressure proximal to the balloon) was derived from the Finometer pressure waveform, and HR was determined from the electrocardiogram. FBF, BAP, MAP, CO, and HR were determined by averaging values during the last 30 s of rest, exercise, exercise with inflation, exercise following deflation, and recovery. In addition, all values were analyzed and averaged during the first 10 s of target balloon inflation (nadir) and the first 10 s immediately following balloon deflation. FVC was calculated as (FBF/BAP) × 100 and expressed as ml min^−1^ (100 mmHg)^−1^.

All values are expressed as means ± SE. Within a given protocol, the FBF, FVC, BAP, systemic MAP, HR, and CO during rest, exercise, the nadir after balloon inflation, exercise at the end of the balloon inflation, exercise following deflation, and recovery were analyzed by repeated measures analysis of variance (ANOVA). When significance was detected, Tukey’s *post hoc* test was used to identify individual differences and adjust *P* values to account for multiple comparisons, to preserve an overall type I error rate of 0.05.

Percent recovery in FBF and FVC were calculated (steady state inflation plus exercise value − nadir)/[steady state exercise (control) value − nadir]. To investigate the contribution of α-adrenergic vasoconstriction on percentage recovery of blood flow and conductance, paired *t*-tests were performed between drug conditions (with and without phentolamine). To further explore the contribution of local vasodilation to any restoration of flow, we analyzed balloon resistance and forearm vascular resistance and considered them individually and in series (O’leary and Sheriff, 1995; Casey and Joyner, 2009a,b). Using systemic arterial pressure (SAP; Finometer), brachial artery pressure distal to the balloon (BAP; catheter) and brachial artery blood flow, we calculated the resistance of the balloon (SAP − BAP/flow) and vascular resistance (BAP/flow). The total resistance was calculated as the sum of these two resistors. Changes in vascular and balloon resistance were analyzed from the onset of balloon inflation (nadir) until the end of the inflation period and expressed as a percentage change. One way repeated measures ANOVA were used to compare the percentage change in resistance between drug conditions. Statistical significance was set *a priori* at *P* < 0.05. Statistical analyses were performed with SigmaStat 2.03 (SPSS, Inc.).

## Results

Eight of the nine subjects completed both of the exercise trials. One subject did not complete the entire protocol due to technical difficulties associated with the balloon during the phentolamine trial and was excluded from the analysis. Those subjects included in the group analysis were 29 ± 2 years of age, 180 ± 2 cm in height, and weighed 82 ± 4 kg (BMI: 25 ± 1 kg m^−2^).

### Forearm blood flow and vasodilation during exercise with balloon inflation

**Figure 2:**
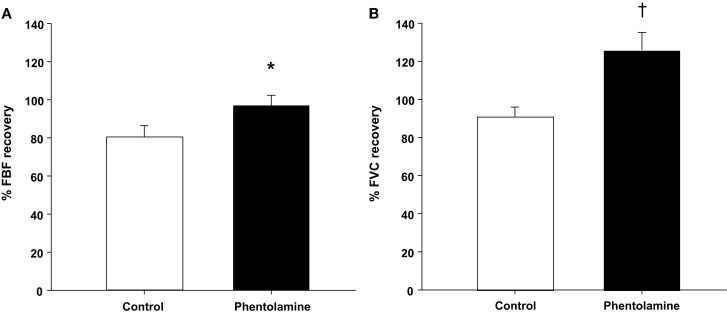
**Percentage recovery in forearm blood flow [FBF; (A)] and forearm vascular conductance [FVC; (B)] during balloon inflation**. The percentage recovery in FBF and FVC was enhanced under local α-adrenergic blockade during exercise with hypoperfusion. *P < 0.05 vs. control (no drug); ^†^*P* < 0.01 vs. control (no drug).

Group mean data for FBF and FVC responses are presented in Table 1. As expected, exercise increased FBF and FVC in both exercise trials (*P* < 0.01). Balloon inflation (nadir) during the exercise trial with no drug acutely reduced FBF by 38% and FVC by 26% (*P* < 0.001). FBF and FVC at the end of inflation were partially restored to exercise (control) levels, which were substantially higher than their respective nadir values (*P* < 0.001). The percentage recovery of FBF and FVC during the exercise trials are presented in Figures 2A,B.

**Table 1:** **Forearm blood flow and vasodilation during exercise with balloon inflation**.

	Baseline	Exercise (control)	Inflation (nadir)	Inflation (steady state)	Deflation (acute)	Deflation (steady state)
**20% MVC (NO DRUG)**						
FBF (ml min^−1^)	95 ± 25	468 ± 33*	298 ± 31*^†^	436 ± 37*^‡^	509 ± 43*^‡^^§^	501 ± 37*^‡^^§^
FVC [ml min^−1^ (100 mmHg)^−1^]	107 ± 27	520 ± 42*	385 ± 28*^†^	510 ± 44*^‡^	558 ± 56*^‡^	558 ± 47*^‡^
**20% MVC (PHENTOLAMINE)**						
FBF (ml min^−1^)	328 ± 36^a^	657 ± 70*^a^	383 ± 49^†b^	642 ± 70*^‡^^a^	755 ± 88*^‡^^§^^a^	685 ± 75*^‡^^a^
FVC [ml min^−1^ (100 mmHg)^−1^]	379 ± 45^a^	737 ± 85*^a^	544 ± 62*^†^^a^	782 ± 91*^‡^^a^	839 ± 101*^‡^^a^	744 ± 88*^‡^^a^

Values are means ± SE. FBF, forearm blood flow; FVC, forearm vascular conductance; MVC, maximal voluntary contraction. *P < 0.01 vs. baseline; ^†^*P* < 0.001 vs. exercise (control); ^‡^*P* < 0.001 vs. nadir; ^§^*P* < 0.05 vs. inflation (steady state); ^a^*P* < 0.01 vs. no drug trial; ^b^*P* < 0.05 vs. no drug trial.

### Impact of α-adrenergic receptor blockade on blood flow recovery during hypoperfusion

Infusion of phentolamine increased FBF and FVC throughout the entire exercise trial compared to the control (no drug) trial (*P* < 0.01–0.05; Table 1). Balloon inflation (nadir) during the exercise trial with phentolamine acutely reduced FBF by 45% and FVC by 28% (*P* < 0.001). The FBF and FVC at the end of inflation under α-adrenergic receptor blockade were greater than the values observed during the no drug trial (*P* < 0.01; Table 1). Consequently, the percentage recovery of FBF and FVC during the trial with phentolamine were substantially greater than the percentage recovery values observed during the no drug trial (Figures 2A,B). The time to reach steady state FBF during balloon inflation was similar between the no drug and phentolamine trials (49 ± 5 vs. 47 ± 4 s; *P* = 0.48).

Acute balloon inflation caused similar increases in absolute (0.06 ± 0.01 vs. 0.05 ± 0.01 mmHg ml min^−1^; *P* = 0.17) and relative (31 ± 2% vs. 35 ± 5%; *P* = 0.41) vascular resistance between the no drug and phentolamine trials. Vascular resistance during balloon inflation (from nadir to end of inflation) decreased during the no drug (0.27 ± 0.02 vs. 0.21 ± 0.02 mmHg ml min^−1^; *P* < 0.001), and phentolamine (0.20 ± 0.02 vs. 0.14 ± 0.02 mmHg ml min^−1^; *P* < 0.01) trials. Although the absolute reduction in vascular resistance was similar between trials (*P* = 0.76), the percentage reduction in vascular resistance was greater with phentolamine (−31 ± 2% vs. −24 ± 2%; *P* < 0.01 vs. no drug trial). Balloon resistance decreased (from nadir to end of inflation) in the no drug (0.05 ± 0.01 vs. 0.02 ± 0.01 mmHg ml min^−1^; *P* < 0.01) and phentolamine (0.05 ± 0.01 vs. 0.03 ± 0.01 mmHg ml min^−1^; *P* < 0.01) trails. However the absolute (−0.03 ± 0.01 vs. −0.03 ± 0.01; *P* = 0.93) and relative (−50 ± 8% vs. −49 ± 4%; *P* = 0.91) changes in balloon resistance were not different between drug conditions.

### Effect of α-adrenergic receptor blockade on vascular responses to exogenous tyramine

**Figure 3:**
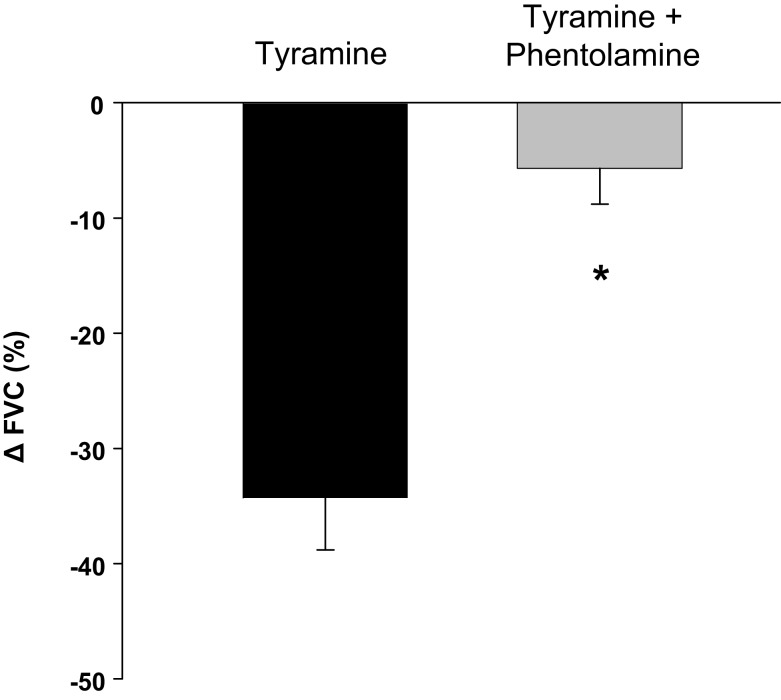
**Effectiveness of phentolamine in blocking α-adrenergic vasoconstriction**. α-Adrenergic blockade via phentolamine substantially reduced the vasoconstrictor responsiveness (Δ forearm vascular conductance; FVC) to tyramine. *P < 0.001 vs. control (no drug).

Tyramine caused a substantial reduction in FVC (87 ± 26 vs. 124 ± 25 ml min^−1^ (100 mmHg) ^−1^; *P* < 0.001). α-Adrenergic receptor blockade with phentolamine prevented a significant vasoconstrictor response to tyramine (300 ± 33 vs. 318 ± 34 ml min^−1^ (100 mmHg)^−1^; *P* = 0.16). The relative tyramine-induced reductions in FVC (with and without phentolamine) are presented in Figure 3.

### Vasoconstrictor responsiveness and blood flow recovery during hypoperfusion

**Figure 4:**
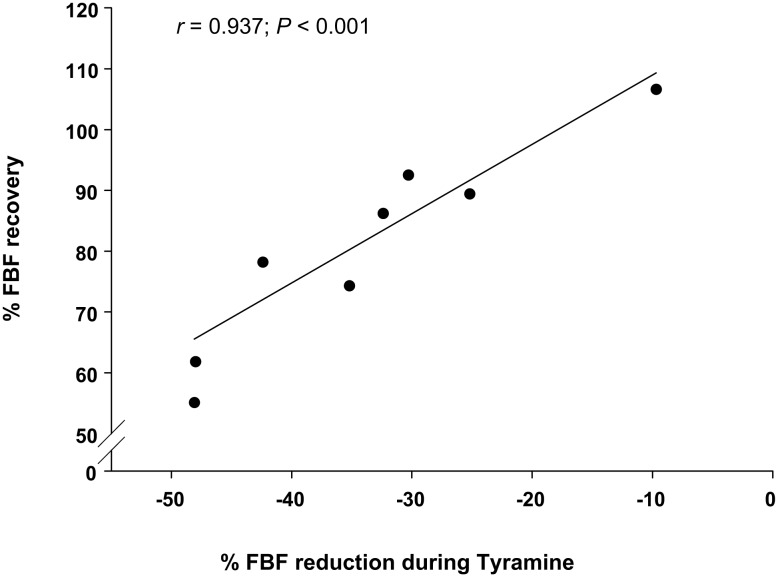
**Relationship between vasoconstrictor responsiveness and percentage recovery in forearm blood flow (FBF) during hypoperfusion**. Vasoconstrictor responsiveness to endogenous norepinephrine release via tyramine (percentage reduction in forearm blood flow; FBF) is related to percentage recovery of FBF during balloon induced hypoperfusion under control (no drug) conditions.

Figure 4 illustrates a strong relationship between the vasoconstrictor responsiveness to endogenous norepinephrine release via infusion of tyramine and the percentage FBF recovery during forearm exercise with hypoperfusion. Subjects with greater vasoconstrictor responsiveness to tyramine (i.e., greater reduction in FBF) demonstrated a lower percentage FBF recovery.

### Hemodynamic changes

Systemic hemodynamic responses during exercise are presented in Table 2. Exercise resulted in an increase in MAP in both control (no drug) and phentolamine trials (*P* < 0.05). MAP remained elevated above baseline values throughout each trial (*P* < 0.05). Estimated CO did not change with exercise (control) compared to baseline in either trail, despite a slight increase in HR during the control (no drug) trial. Compared to baseline values CO was elevated during balloon inflation and deflation in the control (no drug trial), whereas it was only elevated during the deflation period in the phentolamine trial (*P* < 0.05). Important to the present study, MAP, HR, and CO did not change with balloon inflation compared to exercise (control) values in either trail.

**Table 2:** **Systemic hemodynamic responses (*n* = 8)**.

	Baseline	Exercise (control)	Inflation (nadir)	Inflation (steady state)	Deflation (acute)	Deflation (steady state)
**20% (CONTROL)**						
Mean arterial pressure (mmHg)	93 ± 3	98 ± 4*	99 ± 4*	100 ± 4*	100 ± 4*	99 ± 4*
Brachial artery pressure (mmHg)	90 ± 2	91 ± 3	79 ± 3*^‡^	86 ± 2^†^^‡^	91 ± 2^†^	89 ± 2^†^
Heart rate (beats min^−1^)	66 ± 2	68 ± 2*	68 ± 2*	68 ± 2*	68 ± 2*	68 ± 2*
Cardiac output (l min^−1^)	4.8 ± 0.3	5.2 ± 0.4	5.3 ± 0.4*	5.3 ± 0.3*	5.4 ± 0.4*	5.5 ± 0.4*
**20% (PHENTOLAMINE)**						
Mean arterial pressure (mmHg)	93 ± 4	97 ± 3*	98 ± 3*	100 ± 3*	99 ± 3*	101 ± 3*
Brachial artery pressure (mmHg)	88 ± 2	90 ± 2	70 ± 2*^‡^	78 ± 3*^†^^‡^	90 ± 2^†^	91 ± 2^†^
Heart rate (beats min^−1^)	67 ± 2	68 ± 2	68 ± 2	68 ± 2	68 ± 2	68 ± 2
Cardiac output (l min^−1^)	5.1 ± 0.3	5.4 ± 0.4	5.5 ± 0.4	5.5 ± 0.5	5.6 ± 0.5*	5.7 ± 0.5*

Values are means ±  SE; *P < 0.05 vs. baseline; ^†^*P* < 0.05 vs. nadir; ^‡^*P* < 0.05 vs. exercise (control).

## Discussion

The novel findings of our study are (1) α-adrenergic blockade unmasks a greater compensatory vasodilation and flow recovery to hypoperfused contracting muscle, (2) the initial increase in vascular resistance at the onset of balloon inflation does not appear to be α-adrenergic mediated, and (3) vasoconstrictor responsiveness to endogenous norepinephrine is related to a lower flow recovery during exercise with hypoperfusion.

Stimulation of chemosensitive afferents in contracting muscle can elicit marked increases in MSNA to resting and active muscle (Mark et al., 1985; Savard et al., 1987; Victor et al., 1987, 1988). The increase in MSNA is further enhanced in hypoperfused and/or partially ischemic contracting human muscle (Michikami et al., 2000; Daley et al., 2003). Theoretically, the enhanced sympathetic drive to the muscle and subsequent vasoconstriction could limit blood flow to the active tissue. In the present study we examined whether α-adrenergic vasoconstriction restrained flow during exercise with hypoperfusion and possibly explained the incomplete compensatory flow response (<100% recovery) commonly observed in our studies (Casey and Joyner, 2009a,b, 2011a,c). Our data demonstrate that α-adrenergic blockade unmasks a substantial vasodilation and restores blood flow to pre-inflation levels when compared to control trials (97% vs. 80% recovery, respectively; Figure 2). These findings suggest that sympathetic outflow during concurrent exercise and hypoperfusion partially limits the compensatory vasodilation in the human forearm.

Interestingly, there is an absence of a significant pressor response (above exercise alone) in our model of hypoperfusion (Table 2), thus suggesting that balloon inflation and subsequent hypoperfusion likely did not result in greater sympathetic outflow when compared to free flow exercise conditions. Therefore, it may be possible that the α-adrenergic restraint observed with hypoperfusion may be due to an exaggerated α-adrenergic receptor responsiveness in the partially ischemic tissue. Along these lines, the relative vasoconstriction produced by α-adrenergic activity in the coronary circulation of dogs during exercise is greater during hypoperfusion (Laxson et al., 1989) than during normal arterial inflow (Bache et al., 1988). It is also possible that an attenuated functional sympatholysis might exist in the contracting muscle when blood flow and oxygen delivery are compromised. However, previous data form our group did not provide any evidence for reduced vasoconstrictor responsiveness (augmented functional sympatholysis) during hypoxic exercise (Wilkins et al., 2006).

Similar to our previous studies, vascular resistance increased at the onset of balloon inflation. The findings of the present study suggest that an enhanced α-adrenergic vasoconstriction at the onset of balloon inflation does not explain the initial increase in vascular resistance. In this context, α-adrenergic blockade did not prevent the initial rise in vascular resistance when expressed as absolute or relative changes. Although the reasons for the acute increase in vascular resistance at the onset of balloon inflation in our model of hypoperfusion are not completely clear it may be related to dampening of pulsatile flow by balloon inflation. Along these lines, pulsatile flow has been suggested to be a critical component for the release of endothelium-derived vasodilators (Rubanyi et al., 1986). Another possible explanation is that a sudden drop in perfusion pressure causes resistance vessels to recoil before autoregulatory vasodilation, a response that has been observed during mild exercise in dogs (Koch et al., 1991). Lastly, alterations in vascular bed compliance at the onset of balloon inflation may also contribute to the acute increase in vascular resistance observed in our model of hypoperfusion (Zamir et al., 2007).

In the current study we observed an extremely strong relationship between the vasoconstrictor responsiveness to endogenous norepinephrine release (via tyramine) and ability to restore blood flow to the contracting muscle during exercise with hypoperfusion (Figure 4). That is an individual with greater vasoconstrictor responses to endogenous norepinephrine release demonstrated a blunted recovery of flow during the period of exercise with balloon inflation. This relationship suggests that the α-adrenergic tone of the resistance vasculature in the forearm may play an important role in the ability to compensate and restore blood flow to underperfused skeletal muscle during exercise.

### Experimental considerations

The use of a non-specific α-adrenergic antagonist (phentolamine) in the current study did not allow us to discern the relative roles of α_1_- and α_2_-adrenergic receptors in the restriction of compensatory vasodilation during forearm exercise with hypoperfusion. In young healthy men α_2_-adrenergic receptors have a greater contribution to basal forearm vascular tone compared to α_1_-adrenergic receptors (Dinenno et al., 2002b). However, in the coronary circulation of dogs, α_1_- but not α_2_-adrenergic mediated vasoconstriction limits blood flow distal to a coronary artery stenosis (Laxson et al., 1989). Therefore, it is unclear whether α_1_ and α_2_-adrenergic mechanisms contribute differently to the vasoconstrictor restraint of flow during exercise with hypoperfusion in humans.

Administration of phentolamine altered baseline blood flow and the absolute blood flow responses to the exercise. It could be argued that these changes in flow before balloon inflation may explain the enhanced flow recovery following α-adrenergic blockade. However, the use of percent recovery (steady state inflation plus exercise value − nadir)/[steady state exercise (control) value − nadir] in the comparison between drug trials clearly accounts for the differences in flow before balloon inflation. Additionally, the reduction in flow caused by balloon inflation still evoked compensatory vasodilation suggesting that a metabolic error signal was present in spite of the higher flow.

Our series of experiments (Casey and Joyner, 2009a,b, 2011a,c) have consistently demonstrated that there is only a partial recovery of FBF (<100%) during exercise with acute hypoperfusion and this response is variable between subjects (see Figure 4 from Casey and Joyner, 2011b). However, the percentage FVC recovery tends to be greater than the percentage FBF recovery and in some cases reaches and/or exceeds 100% (Casey and Joyner, 2011a). In the current study the FVC recovery during the control trial was 91%, which was less than previously reported (Casey and Joyner, 2011a,c). The discrepancies in percentage FVC recovery between studies might be related to the extent of collateral channels in the forearm and magnitude of the restoration of distal perfusion pressure in the subjects of each study. Despite the somewhat lower percentage FVC recovery under control conditions in the current study there was a substantial improvement during phentolamine administration and supports the notion that α-adrenergic vasoconstriction limits compensatory vasodilation and flow recovery to hypoperfused contracting muscle.

## Conclusion

This study demonstrates that α-adrenergic mediated vasoconstriction restricts compensatory vasodilation and flow during forearm exercise with hypoperfusion. However, the initial rise in vascular resistance at the onset of hypoperfusion via balloon inflation is not explained by an enhanced α-adrenergic vasoconstriction. Taken together our findings suggest that α-adrenergic mediated vasoconstriction plays an important role in the “less than perfect” compensatory flow response. Moreover, our data raise the concern that patients with enhanced α-adrenergic vasoconstrictor responsiveness might be more at risk to ischemia during exercise, especially in vascular regions distal to a stenosis.

## Conflict of Interest Statement

The authors declare that the research was conducted in the absence of any commercial or financial relationships that could be construed as a potential conflict of interest.
